# The Role of Heme Oxygenase-1 as an Immunomodulator in Kidney Disease

**DOI:** 10.3390/antiox11122454

**Published:** 2022-12-13

**Authors:** Virginia Athanassiadou, Stella Plavoukou, Eirini Grapsa, Maria G. Detsika

**Affiliations:** 1 Department of Nephrology, School of Medicine, National and Kapodistrian University of Athens, Aretaieion University Hospital, 11528 Athens, Greece; 2 1st Department of Critical Care Medicine & Pulmonary Services, GP Livanos and M Simou Laboratories, Evangelismos Hospital, National and Kapodistrian University of Athens, 10675 Athens, Greece

**Keywords:** heme oxygenase, kidney, immune injury

## Abstract

The protein heme oxygenase (HO)-1 has been implicated in the regulations of multiple immunological processes. It is well known that kidney injury is affected by immune mechanisms and that various kidney-disease forms may be a result of autoimmune disease. The current study describes in detail the role of HO-1 in kidney disease and provides the most recent observations of the effect of HO-1 on immune pathways and responses both in animal models of immune-mediated disease forms and in patient studies.

## 1. Introduction

Heme oxygenase (HO)-1 has been linked to the regulations of immunological and pathophysiological processes, such as inflammation, apoptosis and cytoprotection, mainly through its downstream effector molecules, carbon monoxide, bilirubin and biliverdin. Inflammation and autoimmune diseases are key factors for kidney disease. In the kidney, glomerular capillaries may become targets of inflammation, ultimately leading to severe and irreversible tissue injury. The involvement of HO-1 in immune-mediated forms of kidney injury has been studied extensively. The current study describes in detail the potential role of HO-1, in various forms of kidney injury, as a mediator of immunological mechanisms that drive disease manifestation and progression.

**Figure 1:**
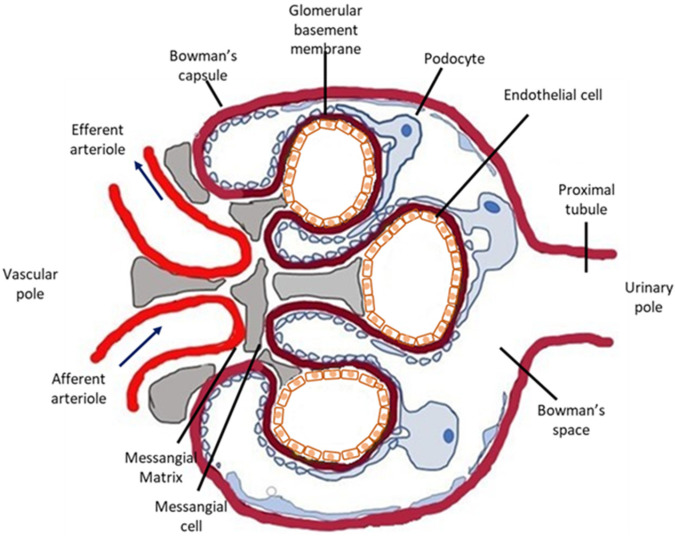
Schematic representation of the glomerulus. The glomerulus is a cluster of capillaries within the Bowman’s capsule. Three different cell types comprise the glomerulus: endothelial cells, epithelial cells (podocytes) and mesangial cells. Endothelial cells are fenestrated, while mesangial cells and the mesangial matrix support the glomerular capillaries. Podocytes are located on the urinary side of the glomerular basement membrane, and they have long foot processes that wrap around the glomerular capillaries.

The various forms of kidney injury usually occur due to injury of the renal glomerulus. Due to their highly specialized structure and function, renal glomeruli are known to be particularly vulnerable to injury. Briefly, the glomerulus (Figure 1) is a tri-cellular structure surrounded by the glomerular (Bowman’s) capsule. Endothelial cells line the luminal side of the glomerular basement membrane (GBM); epithelial cells, also known as podocytes, are anchored on the outer surface of the GBM; and mesangial cells support the capillary loops. Glomeruli form a complex microvascular bed, the glomerular tuft, that functions as a highly selective plasma filter while retaining high-molecular-weight molecules and cells in circulation (Figure 1).

## 2. HO-1 and IgA Nephropathy

IgA nephropathy is a form of chronic glomerulonephritis characterized by the deposition of IgA immune complexes in glomeruli. It is the most common form of glomerulonephritis worldwide [1]. The majority of cases are idiopathic, but in recent years, secondary forms of the disease have appeared after various infections (Haemophilus parainfluenzae, HIV, cytomegalovirus) [2].

Symptoms include the onset of macroscopic hematuria usually one or two days after a febrile infectious episode, thus mimicking infectious glomerulonephritis. Urine analysis of patients with IgA has detected the presence of deformed red blood cells and sometimes red blood cell casts. Mild proteinuria (<1 g/day) is also typical and may occur without hematuria, while serum creatinine levels are usually normal at diagnosis [3].

The pathogenic mechanism that causes IgA nephropathy remains unknown, but accumulated evidence has led to the “four-hit hypothesis”, starting with an abnormal glycosylation pattern of IgA (galactose-deficient IgA1) manifested through increased levels of poorly O-galactosylated IgA1 (gd-IgA1) in blood circulation, which causes the production of circulating auto-antibodies and consequentially the formation and deposition of immune complexes in the mesangium [4,5].

IgA nephropathy diagnosis is confirmed with renal biopsy and immunofluorescence methodology, which reveal granular IgA and complement factor 3 (C3) deposits located in the mesangium with foci of proliferative or necrotic segmental lesions. However, mesangial IgA deposits are considered non-specific and may be detected in many other disorders, such as immunoglobulin A-related vasculitis, HIV infection, psoriasis, lung cancer and several other disorders of the connective tissue. Examination of kidney tissue sections, obtained from IgA patients, under an electron microscope showed increased cellularity and an increased matrix in the mesangium, endocapillary proliferation of neutrophils and subendothelial deposits. Finally, normal levels of complement factors are detected with immunoassays, while an elevated IgA plasma concentration may sometimes be detected with serum electrophoresis [6].

Nitric oxide (NO) and advance oxidation protein products (AOPP) are strong markers of oxidative stress, and their elevated concentrations have been determined in serum samples of patients with severe IgA glomerulopathy [7]. Nakamura et al. compared patients who suffered from IgA nephritis with healthy controls, revealing that exposure to oxidative stress in IgA was of detrimental importance to the progression of renal injury [8]. This could be due to under-expression of superoxide dismutase (SOD) and the consequent exacerbation of tissue injury due to suppression of reactive oxygen species (ROS) scavenging ability (10). Furthermore, studies have identified a dinucleotide guanosine thymine (GT) repeat polymorphism of the HO-1 gene promoter that results in increased HO-1 expression when the GT length is shorter (S-allele) rather than when it is longer (L-allele). These two different alleles may influence the onsets and progressions of many different renal diseases [9], and several studies have shown a direct association between short (GT)n repeats and a higher induction rate of HO-1, which promotes the progression of IgA nephropathy [10].

**Figure 2:**
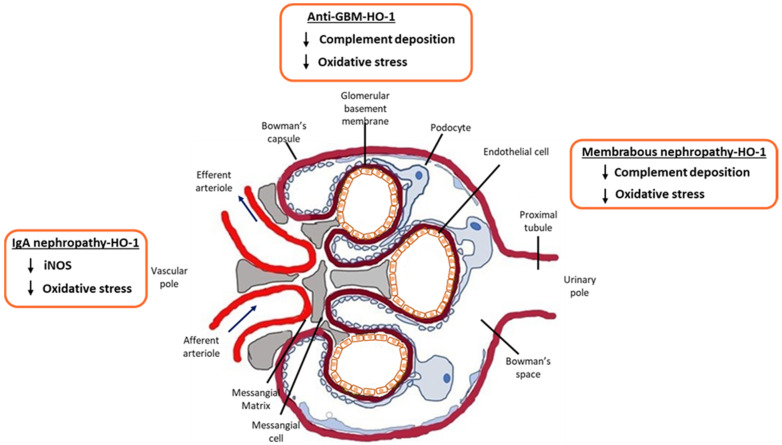
HO-1 immunomodulatory effects in glomerular diseases. Schematic representation of the effect of HO-1 in diseases that affect the glomerulus.

Following intravascular hemolysis, free heme, the natural substrate of HO-1 and a powerful activator the complement cascade, is released. Heme has been reported to activate the alternative complement pathway [11] and may therefore be implicated in complement-mediated renal injury [12]. Free heme influences innate immune responses through the activation of Toll-like receptor 4 and ROS-dependent pathways, which in turn, through complex signaling pathways, promote the expression of proinflammatory cytokines. In addition, heme degradation byproducts (CO, biliverdin, bilirubin) and HO-1 constitute key molecules that upregulate the secretion of anti-inflammatory cytokines, such as IL-10 [13] (Figure 2). On the other hand, many pro-inflammatory enzymes, such as cyclooxygenase-2 (COX-2) and inducible nitric oxide synthase (iNOS), are in fact hemoproteins whose functions may be impaired due to insufficient free heme once that heme has been catalyzed by HO-1 [14]. Micro and/or macroscopic hematuria is a typical symptom of IgA nephropathy that suggests potential induction of HO-1 in the glomeruli [15]. A previous study used various concentrations of hemin to induce glomerular HO-1 expression in vitro and reported that when the concentration of hemin reached a critical level of 200 µM, HO-1 expression started to diminish. This could partly be explained by the fact that various forms of glomerular disease display increased levels of free radicals (OH^−^), hydrogen peroxide (H_2_O_2_) and active Fe^2+^. These may be further augmented by heme degradation of HO; therefore, a HO-1 expression-limiting mechanism may be necessary [16].

The clinical course of IgAN is highly variable. In many cases, the disease goes unnoticed and requires no treatment. The most important parameters that influence disease progression include the degree of proteinuria, uncontrollable hypertension and histopathological lesions on biopsies [17].

Patient assessment is usually performed with the “IgA Nephropathy Prediction Tool” (IIgAN-PT) that uses parameters such as histological findings, eGFR, the degree of proteinuria, the blood pressure value, medication prior to biopsy and demographics (sex, age, ethnicity) in order to estimate the individual five-year risk of renal progression toward end-stage renal disease (ESRD). Independently of IIgAN-PT results, all patients should receive supportive hypertension and proteinuria treatment. Supportive therapy consists of medications that inhibit the renin–angiotensin axon, such as angiotensin receptor blockers (ARB) or angiotensin-converting enzyme inhibitors (ACEi) [18]. These reduce both systemic and intraglomerular blood pressure—and thus glomerular injury due to hypertension—and lower the degree of proteinuria. If a patient’s eGFR is greater than 30 mL/min/1.73 m^2^, the addition of sodium–glucose co-transporter 2 (SGLT2) to their therapy could further reduce proteinuria [19]. Finally, due to toxicity effects, the use of steroids is only supported in cases of rapid declines in renal function and uncontrolled increases in proteinuria [20].

## 3. HO-1 and Membranous Nephropathy (MN)

MN is an autoimmune glomerular disease and a major cause of nephrotic syndrome in the adult population. Symptoms include insidious onset of edema, prominent proteinuria with mild urinary sediment, normal or deteriorating renal function and normal or elevated blood pressure [21]. Spontaneous remission can be seen in about 30% of patients. In most cases (about 70%), MN is reported as idiopathic. In these cases, antibodies against the M-type phospholipase A2 receptor (PLA2R) found in podocytes are linked to that specific locus and form immune complexes in situ that activate the complement membrane attack complex (MAC, C5b-9). However, MN can also develop as secondary MN due to a specific etiological factor. The main causes are solid tumors (lung, colon, rectum, kidney, breast, stomach), autoimmune diseases and microbial infections. In fact, in areas that are endemic of infections, such as malaria or schistosomiasis, the latter will consist of the main cause of MN [21].

The diagnosis of MN is also based on renal biopsy. Optical microscopy reveals the diffuse thickening of the GBM as a reaction to the subepithelial formation of immune complexes. No cellular hyperplasia or inflammatory cells are detected. The glomerular basement membrane thickens in a homogeneous way but gradually creates protrusions into the subepithelial space that eventually embrace and integrate the immune complexes into it. Immunofluorescence detects granular deposits of IgG and C3 along the basement membrane, while electron microscopy uncovers diffuse effacement of podocyte foot processes, as well as dense deposits in the subepithelial area [21].

Heymann et. al. described for the first time an experimental rat model of MN in which the pathogenic role of immune complexes was confirmed [22]. Heymann nephritis (HN) was induced through injections of proximal tubule brush border antigens (active HN) or their corresponding antibodies (passive HN) into rats. The autoantigen target in HN is megalin, a transmembrane protein located in the brush border of the proximal tubule and on podocyte foot processes in rats [23]. Studies of active and passive HN proved that subepithelial deposits are formed in situ against a constitutional endogenous antigen rather than through a circulating immune complex [24]. The HN model has allowed researchers to identify the important role of complement-mediated cytotoxicity in podocyte injury and proteinuria in this model. Early studies in HN showed co-localization of C3 and C5b-9 to the immune deposits. Furthermore, it was shown that the podocytes that were present in the urine of passive HN rats were coated with C5b-9 [25]. HN progression correlated with persisting urinary excretion of C5b-9, indicating continuous complement activation at the GBM.

Membrane-bound proteins, known as complement regulatory proteins (CRPs), act to eliminate complement activation and therefore MAC formation and lysis. A well-known CRP is the decay-accelerating factor (DAF, CD55). The DAF accelerates the decay of C3 and C5 convertases and thus restricts MAC formation [26]. In rats, other CRPs are Crry and CD59 [26]. Studies of DAF expression in rats have revealed constitutive DAF expression exclusively in podocytes [27]. In order to study the protective role of the DAF in complement-induced podocyte injury, a previous study generated a transgenic rat model of DAF depletion (*Daf*^−/−^) [28]. Histological, clinical, and biochemical examinations (creatinine levels, albuminuria, urine albumin to urine creatinine ratio) in *Daf*^+/+^ and *Daf*^−/−^ rats demonstrated no significant differences prior to administration of complement-fixing antibody anti-Fx1A. Following anti-Fx1A administration, proteinuria levels were significantly elevated in *Daf*^−/−^ rats. Immunofluorescence staining in rats that received anti-Fx1A evidenced greater C3 depositions in *Daf*^−/−^ rats than in *Daf*^+/+^ rats, suggesting a protective role of the DAF in podocyte complement-induced injury [28]. Several studies have demonstrated that HO-1 upregulates the DAF, which in turn reduces complement activation and complement-mediated injury [16]. In that context, HO-1 induction could be a useful tool as a potential treatment strategy against complement-mediated glomerulonephritis via its immunomodulatory effects, including DAF induction.

To explore the previously mentioned properties of HO-1, Wu et al. used an experimental animal model of induced MN in BALB/c mice by introducing intravenously cationic bovine serum albumin and dividing the animals into three groups. The first group was treated with a weekly intraperitoneal administration of cobalt protoporphyrin (CoPP), a HO-1 inducer; the second with tin protoporphyrin (SnPP), an HO-1 inhibitor; and the third with saline. The MN-CoPP group exhibited an HO-1 upregulation and presented a clear improvement of symptoms (a decrease in proteinuria and normalization of the serum-albumin and cholesterol levels). CoPP treatment also significantly reduced production of serum anti-cBSA antibodies. Although immunofluorescence staining remained positive for all three groups, the MN-CoPP group exhibited lesser intensity in the glomerular membrane and reduced C3 depositions in respect to the other two groups, especially the MN-SnPP group. The concentrations of markers of oxidative stress, such as thiobarbituric acid reactive substances (TBARSs), were evaluated both in the serum and the kidneys and found to be notably higher in respect to non-MN mice, but significant differences were assessed between the MN-CoPP group and the MN-SnPP group. CoPP treatment decreased oxidative stress markers both in the serum and the kidneys, suggesting that HO-1 may have an antioxidative effect on a local and on a systemic level [29].

Complement activation seems to induce ROS in podocytes that undergo mitochondrial dysregulation in MN [24]. Pyroptosis is a recently identified type of regulated cell death that follows bacterial or viral infections (40). Complex signaling pathways, including activation of inflammasomes and pro-inflammatory cytokines, lead to activation of caspase-1 and consequently formation of pores on the membranes, resulting in cell death and excess of pro-inflammatory cytokines and ROS [30]. Wang et al. described complement mediated pyroptosis in podocytes with a concurrent mitochondrial depolarization and ROS production. Blocking ROS production reversed complement mediated pyroptosis. Immunohistochemistry of MN glomeruli confirmed the co-localization of pyroptosis-related proteins, such as caspase-1 and gasdermin D (GSDMD), as well as synaptopodin, an actin-associated protein found in podocytes. Furthermore, C3a and C5a promoted overexpression of caspase-1 and GSDMD in the podocytes in vitro and influenced the integrity of cellular membranes and the depolarization of the podocyte mitochondrial membranes. When the podocytes of MN patients were incubated with inhibitors of key pyroptosis molecules, C3a and C5a did not affect podocyte membrane integrity [31].

Recent studies have assessed the role of HO-1 in blocking pyroptosis. Carbon monoxide (CO) as a byproduct of the HO-1 catalysis of hemin seems to block activation of caspase-1 through direct inhibition of its inducer molecule, NLRP3-ASC [32,33]. The NF-E2-related factor (Nrf2) is a transcriptional factor that regulates the cellular antioxidant response to oxidative stress by inducing the expressions of antioxidant and cytoprotective molecules, one of which is HO-1 [34]. Sirtuin is a deacetilase protein essential to the integrity of podocytes’ cytoskeletons [35]. Under stress conditions, sirtuin seems to induce an overexpression of Nrf2 that leads to upregulation of HO-1 expression in podocytes [36]. In a murine renal ischemia/reperfusion (I/R) injury model, Diao et al. investigated the role of the NF-E2-related factor/heme oxygenase-1 (Nrf2/HO-1) as a protective factor against pyroptosis [37]. Protein arginine methylation transferase 5 (PRMT5) is involved in a vast number of physiological and pathological conditions, among them embryonic development, tissue homeostasis and malignancies [38]. PRMT5 is implicated in I/R-induced ROS production. Its inhibition resulted in an upregulation of Nrf2/HO-1, a reduction of oxidative stress markers and a decrease in tissue injury [37].

The highly variable course of MN renders its personalized treatment essential according to the risk of renal impairment progression. Spontaneous remission may occur in about 30% of patients; however, all patients presented with proteinuria should be treated either with an angiotensin receptor blocker (ARB) or an ACEi for three to six months. According to the latest MN treatment guidelines, all patients should be assessed for anti-M-type phospholipase A2 receptor (PLA2R) antibody levels in the blood prior to and during treatment, as an absence of anti PLA2R antibodies in a patient with an initially positive test indicates remission. Corticosteroids plus cyclophosphamide administration, along with supportive therapy, are recommended for patients with severe nephrotic syndrome and a rapid decline of renal function at the onset of the disease [39]. In moderate- to high-risk patients, if remission is not achieved within six months of supportive therapy alone, immunosuppressive treatment with rituximab, calcineurin inhibitors (CNIs) and corticosteroids plus cyclophosphamide may be used unless contraindicated due to severe renal impairment, diffuse interstitial fibrosis or recurrent infections [18].

## 4. HO-1 and Anti-GBM Disease

Anti-GBM disease, formerly known as Goodpasture’s disease, is an infrequent autoimmune type of vasculitis that involves small vessels. It mainly affects the kidneys and the lungs, resulting in complement-induced, rapidly progressive crescentic glomerulonephritis and/or diffuse pulmonary bleeding if not treated promptly [40].

Epidemiological data suggest that anti-GBM affects both genders but at different age spectra. Specifically, there is a clear predominance of anti-GBM in male patients aged between 20 and 40 years and in female patients over 60 years old [40].

Anti-GBM is caused by autoantibodies that are directed against a specific epitope of type IV collagen present in the glomerular basement membrane as well as in the alveolar membrane of the lungs (autoantibodies against the NC1 domain of an α-3 chain of type IV collagen) Genetic predisposition and exposure to certain environmental agents have been postulated to trigger its onset. Various exogenous factors, such as infections; smoking; and drugs, such as alemtuzumab, a monoclonal anti-CD52 antibody used in the therapies of B cell leukemia and relapsing forms of multiple sclerosis, have been implicated. Regarding the genetic background of patients, the related literature demonstrates that there is a correlation between the HLA phenotype and the predisposition to develop the disease when exposed to a causal exogenous factor. In particular, it has been shown that people who present the types HLA DR15 and HLA DR4 show greater predisposition [41].

Anti-GBM diagnosis is confirmed with renal biopsy and immunoassays of anti-GBM antibodies in patients’ serum samples. Characteristic histopathological findings in light microscopy of anti-GBM-diseased kidney sections include intense inflammation with focal segmental and necrotic lesions and the presence of diffuse lunar formations called crescents. Immunofluorescence staining reveals a linear GBM deposition of IgG, mostly of type 1 (IgG1), and, in almost 40% of cases, C3 [40]. Serum creatinine levels at diagnosis are directly correlated with the percentage of crescentic formations in biopsy.

In order to investigate these complicated immune mechanisms, anti-GBM-induced animal models have been vastly developed. In 1962, Steblay et al. were the first to acknowledge that inoculation of human GBM, along with Freund’s adjuvant in sheep, induced crescentic anti-GBM disease [42]. Ryan et al. managed to induce anti-GBM in rats via the administration of a recombinant rat anti-α3 (IV) NC1 antibody, proving that murine experimental models of anti-GBM can be used to assess immune responses that could mirror the immunological responses in humans [43].

All three pathways of the complement cascade (classical, alternative and lectin) seem to be involved in complement-mediated characteristic histopathological lesions, as seen in immunofluorescence of pathological glomeruli both in rats and in humans, leading to the assembly of the complement MAC [44].

As mentioned above, the main immunomodulatory effect of HO-1 is indirect inhibition of MAC via an upregulation of the DAF [16]. Sogabe et al. used glycosylphosphatidylinositol (GPI)-DAF knockout mice to assess the correlation between the complement-regulator DAF and glomerular lesions in experimental anti-GBM nephritis. Renal-tissue biopsy samples from GPI-DAF knockout/anti-GBM-induced and wild-type/anti-GBM-induced mice were examined under optical microscopy. Glomeruli from GPI-DAF knockout mice presented increased mesangium cellularity as well as focal and segmental glomerulosclerosis (FSGS). On the contrary, glomeruli from wild-type mice showed minimal pathological signs at day eight post immunization. Immunofluorescence staining demonstrated linear depositions of IgG along the GBMs in both groups, but only in knockout mice were C3 and fibrinogen deposition observed [45].

Experimentally induced anti-GBM nephritis has provided plenty of evidence of a co-stimulation of cytotoxic enzymes, such as inducible nitric oxide synthase (iNOS), and cytoprotective molecules, such as HO-1 [46]. iNOS and HO-1 are both hemoproteins that, along with other heme-containing molecules, are upregulated by oxidative stress and inflammation. HO-1 activation, as mentioned above, catalyzes free heme, rendering it less available for synthesis of and tampering with the functionality of heme-containing enzymes, thus blocking the formation of oxidative byproducts [47]. On the other hand, NO seems to upregulate production of HO-1 in mesangial cells [48] and in renal tubular epithelial cells [49] suggesting complicated regulatory interactions between the two systems (iNOS and HO-1) and supporting the potential of HO-1 as a target for innovative future therapeutic strategies [50].

Due to the severity of the disease and the poor outcomes if it is left untreated, early treatment should be considered for all patients suspected to be positive for anti-GBM disease and concomitant rapidly progressive glomerulonephritis and/or a pulmonary hemorrhage, even if definite diagnosis through serological tests for anti-GBM antibodies and immunofluorescence is pending. KDIGO guidelines for treatment of anti-GBM suggest concurrent use of immunosuppression with corticosteroids and cyclophosphamide in alternative months, as well as plasmapheresis, except for in patients who need dialysis prior to therapy, are negative for a pulmonary hemorrhage and present 100% cellular crescents in the biopsy. [20]. A recently published retrospective multicenter observational study evaluated the risk for ESRD in patients diagnosed with anti-GBM disease over a period of 20 years and concluded that histopathological findings such as cellular crescents >50% and high creatinine (>4.7 mg/dL) at the onset of the disease are detrimental to renal survival, underlining the need for a more targeted and effective treatment of the disease [51].

## 5. HO-1 and Lupus Nephritis

Systemic lupus erythematosus (SLE) is a complex multisystemic autoimmune disease of unknown etiology that predominantly affects young women. There are a wide range of clinical manifestations and an involute pathogenesis as a result of interactions between genetic, epigenetic, ethnic, immunoregulatory and environmental factors [52]. Lupus nephritis (LN) occurs in about 20–40% of patients with SLE and remains the major risk factor for increased morbidity and mortality, despite advances in its diagnosis and treatment [53].

LN pathogenesis is characterized by multiple interactions between activated immune cells (extra- and intra-renal), the production of autoantibodies and the release of inflammatory mediators. Deposition of immune complexes (ICs) activates the complement cascade within the glomeruli or in the intratubular space, resulting in tissue inflammation [54].

Progress has been made in decoding the roles of innate and adaptive immune cells (particularly neutrophils, monocytes/macrophages, T and B cells) in the pathogenesis of SLE [54]. In particular, the role of monocytes/macrophages in the SLE pathogenesis has been widely studied, yet their exact inflammatory role remains uncertain due to their participation in multiple levels of disease development (phagocytosis, recruitment of other immune cells, cytokine secretion, tissue repair and fibrosis) [55].

Μonocytes from patients with SLE—independently of disease activity—displayed significantly reduced HO-1 levels compared to those of healthy controls, suggesting that this low HO-1 expression and action could contribute to altered monocyte function in SLE and lupus nephritis. [13]. Kishimoto et al. have also demonstrated that glomerular M2-like macrophages from LN patients exhibit lower levels of HO-1 expression. This study showed that a transcriptional HO-1 repressor named Bach-1 can be induced by interferon type I. Bach-1-deficient MRL/lpr mice exhibited high HO-1 expression in kidneys with improved clinical biomarkers and unaltered anti-dsDNA antibody levels [56]. Thus, Bach-1 suggests a potential therapeutic target that could restore the M2-like macrophage function that is linked to increased HO-1 expression and activity [56].

Further studies of LN patients by Cuitino et al. confirm low HO-1 expression in pro-inflammatory monocytes and activated neutrophils with unbalanced function, such as increased phagocytosis and ROS production [57]. Interestingly, cobalt protoporphyrin (Co-PP) seems to induce HO-1 expression with a subsequent modification of LN monocyte phagocytic activity to a level similar to that of healthy controls. Thus, we can speculate that the impaired LN monocyte and neutrophil activity could be in part explained by reduced levels of HO-1 [57]. However, further studies are needed to confirm this hypothesis.

In LN, the primary events are the production of autoantibodies and the glomerular deposition of immune complexes (ICs) that activate complement cascade and immune cells bearing FCγ receptors (FCγRs) [58]. Dendritic cells, T helper cells, B cells and plasma cells all contribute to irregular polyclonal autoimmunity that is mediated with cell-to-cell interactions, immune tolerance and apoptotic mechanisms [58]. Tolerogenic dendritic cells (tolDCs) that are specialized to suppress the immune response may be a promising strategy in SLE treatment [59]. Funes et al. evaluated the therapeutic effect of tolDCs generated with the HO-1 inducer, CoPP, as well as, with dexamethasone and rosiglitazone, in two SLE mouse models [60]. Generation of tolDCs by use of the above agents, showed an efficient tolerogenic profile in vitro but did not improve LN severity or progression although it ameliorated other disease symptoms such as skin lesions [60]. In another study with SLE mice, the administration of hemin, the natural HO-1 substrate and inducer, attenuated disease progression. A marked reduction in proteinuria and glomerular immune complex deposition was observed and a concurrent reduction of inducible NOS expression in the kidney and spleen [61]. Furthermore, a reduction of autoantibodies was also identified. These findings demonstrate a double, anti-inflammatory and immunomodulatory, role of HO-1 induction, highlighting its potential as a novel therapeutic target in LN [61].

Furthermore, CO (a product of heme degradation catalyzed by HO-1) administration can attenuate autoimmunity and prevent clinical disease manifestation in FcγRIIb-deficient mice which is another SLE model [62]. The effects of CO exposure in SLE mice included decreased activated B220^+^CD3^+^CD4^−^T cells in lungs and kidneys, together with low autoantibody levels [63].

In patients with SLE, LN affects both sexes equally, is more severe in children and men and is less severe in older adults. Approximately 10% of patients with LN will progress to ESRD [64], however, this depends on disease classification according to histologic evaluation. The risk for ESRD over 15 years was found to be as high as 44% in class IV LN [65]. SLE patients have a shorter life expectancy compared to those without nephritis and have a standardized mortality ratio of 6 to 6.8, versus the 2.4 ratio in lupus without renal manifestations [66]. An increase of this ratio to 14 is reported for patients with CKD and to 63 for patients with ESRD [67]. However, if LN remission is achieved through treatment, 10 year survival doubles to 95% [68].

Although nephritis may be characterized by clinical symptoms and laboratory markers, renal biopsy is required for confirmation, subclassification, prognosis and management options. The degree and type of glomerular involvement correlate directly with the clinical presentation and guide treatment decisions. LN is currently classified by the International Society of Nephrology (ISN)/Renal Pathology Society (RPS) system, which is based on glomerular histology using light and immunofluorescence microscopy [69]. Treatment recommendations are based on the ISN/RPS biopsy classification. All patients with SLE should be treated with hydroxychloroquine or an equivalent antimalarial unless contraindicated. In general, immunosuppressive therapy of extra renal lupus manifestations is sufficient for class I and II LN. A combination of high-dose corticosteroids plus an immunosuppressive agent is mainly used for patients with active focal proliferative LN (classes IIIA and IIIA/C), active diffuse proliferative LN (classes IVA and IVA/C) or membranous lupus (class V). The treatment of focal or diffuse LN has two phases. The initial or induction therapy with anti-inflammatory and immunosuppressive agent, which addresses acute life- or organ-threatening disease, and the long-term immunosuppressive treatment, which prevents relapses and consolidate remissions. According to the latest guidelines for the management of lupus nephritis, patients with active Class III or IV LN, with or without a membranous component, should be treated initially with glucocorticoids plus either a low-dose intravenous cyclophosphamide or a mycophenolic acid analogue (MPAA). An alternative immunosuppressive regimen that includes a calcineurin inhibitor (CNI) (usually tacrolimus or cyclosporine) with reduced-dose MPAA and glucocorticoids is reserved for patients who cannot tolerate standard-dose MPAA or are unfit for cyclophosphamide-based regimens. There is also an emerging role for B-lymphocyte-targeting biologics in the treatment of LN. Belimumab can be added to standard therapy in treatment of active LN and rituximab may be considered for patients with persistent disease activity or repeated flares. Maintenance therapy would be based on an MPAA regimen or, alternatively, azathioprine. Glucocorticoids should be tapered to the lowest possible dose [18].

## 6. HO-1 and Acute Kidney Injury (AKI)

AKI, characterized by a rapid increase in serum creatinine and/or a decrease in urine output, is common in critically ill patients and is associated with increased morbidity and mortality. The pathophysiology of AKI is complex, involving activation and crosstalk between multiple pathways, including inflammation and oxidative stress. HO-1 regulates oxidative stress, autophagy and inflammation. Furthermore, it controls cell-cycle progression directly and indirectly [70]. Recent studies have revealed that HO-1 expression in monocytes/macrophages may be beneficial, as it was shown to ease the inflammatory response in AKI [13]. HO-1-expressing macrophages were shown to possess a tendency towards an M2 phenotype polarization that contributes to upregulation of anti-inflammatory cytokine (IL-10) expression, suppression of pro-inflammatory cytokine (TNFα) secretion and expression of reparative genes that are beneficial to tissue recovery following AKI [71,72]. Furthermore, the cytoprotective effects of HO-1 attributed to heme degradation byproducts may also exert modulatory effects on AKI [73]. CO was shown to exhibit strong anti-proliferative effects on T cells via IL-2 downregulation, which diminishes inflammation [74]. HO-1 cytoprotective properties were first identified in the kidney in a model of heme-protein-induced AKI [75]. Various studies have confirmed the great potential of HO-1 induction, both pharmacologically and genetically, to regulate immune responses against AKI [71]. However, well-established modes for HO-1 upregulation, in terms of tolerance and efficacy, are essential to initiate the translation of its therapeutic potential into effective patient therapies for AKI.

## 7. HO-1 in Renal Ischemia/Reperfusion Injury (IRI)

IRI is a pathological condition characterized by an initial restriction of blood supply to an organ and a subsequent restoration of perfusion and re-oxygenation [76]. It involves activation of cell death programs, endothelial dysfunction, transcriptional reprogramming and activation of the immune system [77]. IRI is one of the most common causes of AKI. Current knowledge of the role of HO-1 in IRI-induced renal disease is largely based on experience with animal models of kidney disease. Chemical inhibition of HO-2 and HO-1 activity in the healthy kidney results in reduced medullary blood flow, thus supporting the role of HO-1 in the maintenance of medullary perfusion under physiological conditions. A reduction of IRI severity via HO-1 has also been demonstrated with the use of HO-1 chemical inducers [78]. However, the exact underlying mechanisms by which HO-1 exerts its protective effect against IRI remain unknown. In an attempt to elucidate the molecular mechanisms of the cytoprotective effect of HO-1 in IRI, a previous study utilized a mouse model of IRI in HO-1^+/−^ mice and then measured the levels of blood urea nitrogen (BUN) and serum creatinine (SCr). Furthermore, that study investigated the severity of histological changes, as well as HO-1 and vascular cell adhesion molecule 1 (VCAM-1) protein expression levels, inflammatory factor expression and the effects of VCAM-1 blockades. That study reported elevated levels of VCAM-1 expression in HO-1^+/−^ mice during IRI and an increase in the extent of renal-tissue damage and activation of the inflammatory response [79]. Another study imported an experimental model of repeated episodes of IR with 10-day intervals and found that it induced long-term renal protection accompanied by HO-1 overexpression and an increase in M2-macrophages. The aforementioned study investigated the transition between AKI and CKD and involved AKI induction via a single bilateral IR episode (1IR) or three episodes of IR separated by 10 day intervals (3IR) of mild (20 min) or severe (45 min) ischemia [80].

IRI also plays an important role in kidney transplantation and affects delayed renal function after transplantation. The protective role of HO-1 in IRI-mediated injury during kidney transplantation was shown in a recent study that utilized a myeloid-restricted mouse model of deletion of HO-1 (HO-1^M-KO^). IRI in the HO-1^M-KO^ mice resulted in significant renal histological damage, pro-inflammatory responses, and oxidative stress 24 h after reperfusion. An assessment of the animals at a following time point of seven days afterward revealed that the HO-1^M-KO^ mice displayed impaired tubular repair and increased renal fibrosis [81]. Furthermore, the same study showed that hemin mediated HO-1 induction in WT mice, resulting in HO-1 upregulation within the CD11b^+^ F4/80^lo^ subset of the renal myeloid cells [81]. The findings supported the increasing potential of HO-1 as a target of therapeutic strategies in the field of kidney transplantation.

## 8. HO-1 Polymorphisms and Kidney Disease

As mentioned before, there is a direct association between short (GT)n repeats and a higher induction rate of HO-1 and progression of IgA nephropathy [10]. The HO-1 genotype is a risk factor for renal impairment of IgA nephropathy at diagnosis, which is a strong predictor of mortality [10]. Another study investigated the (GT)n short repeat genotype, which promotes induction of HO-1, in renal transplantation and allograft rejection. The study reported that the beneficial effect of the HO-1 genotype was attributed to the donor genotype and not to the recipient when an allograft was exposed to prolonged cold ischemia. Furthermore, allografts from L-donors resulted in more rejections, whereas kidneys that bared the S-allele were less susceptible to injury, thus resulting in reduced rates of allograft rejection [82].

## 9. Clinical Applications

Manipulation of HO-1 expression/activity for potential therapeutic strategies has already been explored. HO-1 may be activated through a wide range of both naturally occurring and chemically synthesized compounds. The most widely used HO-1 inducers/inducers are metalloporphyrins (MPs). These are all heme analogues that differ mainly in the metal moiety of the porphyrin structure. However, numerous other naturally occurring compounds have been reported to induce HO-1, including curcumin, resveratrol, quercentin, carnosic acid, carnosol and anthocyanin [83]. Both MPs, as well as all of the other naturally occurring compounds, have been proposed for their use in the treatments of various immune-mediated diseases, including kidney disease [84] as well as other immune-mediated diseases, such as multiple sclerosis (MS), type 1 diabetes, rheumatoid arthritis, lupus and inflammatory bowel disease [83]. The therapeutic potential of HO-1 has also been highlighted by other studies that have proposed the potential use of MPs with non-immune-mediated forms of disease, such as non-alcoholic fatty liver disease [85]. Finally, manipulation of the HO reaction has also enabled research towards potential therapeutic strategies via CO release via CO-releasing molecule (CORM) administration in preclinical models of type 1 diabetes [86] and in MS [87,88], as well as in models of autoimmune hepatitis [89].

## 10. Conclusions

Apart from its established role as a strong antioxidant and anti-apoptotic enzyme, HO-1 is by now also recognized as an important modulator of various immune pathways and responses. Furthermore, its increased inducibility through multiple varied types of inducers renders it a highly interesting target for novel therapeutic strategies against immune-mediated diseases, including kidney disease. Further research is needed, in order to unravel the exact mechanisms of immunomodulation in kidney disease, which will enable translation into innovative treatment strategies against immune-mediated kidney disease.
